# Association of Postpartum Maternal Mood With Infant Speech Perception at 2 and 6.5 Months of Age

**DOI:** 10.1001/jamanetworkopen.2022.32672

**Published:** 2022-09-21

**Authors:** Gesa Schaadt, Rachel G. Zsido, Arno Villringer, Hellmuth Obrig, Claudia Männel, Julia Sacher

**Affiliations:** 1 Department of Education and Psychology, Freie Universität Berlin, Berlin, Germany; 2 Department of Neuropsychology, Max Planck Institute for Human Cognitive and Brain Sciences, Leipzig, Germany; 3 Department of Neurology, Max Planck Institute for Human Cognitive and Brain Sciences, Leipzig, Germany; 4 Emotion & Neuroimaging Lab, Max Planck Institute for Human Cognitive and Brain Sciences, Leipzig, Germany; 5 Clinic for Cognitive Neurology, Medical Faculty, University Leipzig, Leipzig, Germany; 6 Department of Audiology and Phoniatrics, Charité–Universitätsmedizin Berlin, Berlin, Germany; 7 Department of Psychiatry, Psychotherapy and Psychosomatics, Helios Park Hospital Leipzig, Leipzig, Germany

## Abstract

**Importance:**

Language development builds on speech perception, with early disruptions increasing the risk for later language difficulties. Although a major postpartum depressive episode is associated with language development, this association has not been investigated among infants of mothers experiencing a depressed mood at subclinical levels after birth, even though such a mood is frequently present in the first weeks after birth. Understanding whether subclinical depressed maternal mood after birth is associated with early language development is important given opportunities of coping strategies for subclinical depressed mood.

**Objective:**

To examine whether depressed maternal mood at subclinical levels 2 months after birth is associated with infant speech perception trajectories from ages 2 to 6.5 months.

**Design, Setting, and Participants:**

In this longitudinal cohort study conducted between January 1, 2018, and October 31, 2019, 46 healthy, monolingual German mother-infant dyads were tested. The sample was recruited from the infants database of the Max Planck Institute for Human Cognitive and Brain Sciences. Initial statistical analysis was performed between January 1 and March 31, 2021; the moderation analysis (results reported herein) was conducted between July 1 and July 31, 2022.

**Exposures:**

Mothers reported postpartum mood via the German version of the Edinburgh Postnatal Depression Scale (higher scores indicated higher levels of depressed mood, with a cutoff of 13 points indicating a high probability of clinical depression) when their infants were 2 months old.

**Main Outcomes and Measures:**

Electrophysiological correlates of infant speech perception (mismatch response to speech stimuli) were tested when the infants were aged 2 months (initial assessment) and 6.5 months (follow-up).

**Results:**

A total of 46 mothers (mean [SD] age, 32.1 [3.8] years) and their 2-month-old children (mean [SD] age, 9.6 [1.2] weeks; 23 girls and 23 boys) participated at the initial assessment, and 36 mothers (mean [SD] age, 32.2 [4.1] years) and their then 6.5-month-old children (mean [SD] age, 28.4 [1.5 weeks; 18 girls and 18 boys) participated at follow-up. Moderation analyses revealed that more depressed maternal subclinical postpartum mood (mean [SD] Edinburgh Postnatal Depression Scale score, 4.8 [3.6]) was associated with weaker longitudinal changes of infants’ electrophysiological brain responses to syllable pitch speech information from ages 2 to 6.5 months (coefficient: 0.68; 95% CI, 0.03-1.33; *P* = .04).

**Conclusions and Relevance:**

The results of this cohort study suggest that infant speech perception trajectories are correlated with subclinical depressed mood in postpartum mothers. This finding lays the groundwork for future research on early support for caregivers experiencing depressed mood to have a positive association with children’s language development.

## Key Points

**Question:**

Is subclinical postpartum depressed maternal mood associated with how the young infant’s brain processes speech information?

**Findings:**

In this population-based cohort study, more depressed maternal mood at 2 months after birth was associated with a statistically significant smaller change in infants’ electrophysiological brain responses to syllable pitch perception between ages 2 and 6.5 months.

**Meaning:**

This study’s findings suggest an association between aberrant maturation patterns of infant speech perception and postpartum depressed maternal mood at subclinical levels.

## Teaser

This cohort study examines whether depressed maternal mood at subclinical levels 2 months after birth is associated with infant speech perception trajectories from ages 2 to 6.5 months.

## Introduction

Early human infancy is a critical period for studying language development, as infants already show perceptual capacities for linguistic input.^1,2,3^ Language development hinges on social interaction, with the primary caregiver’s mood affecting communication with the infant. Clinical studies on postpartum depression^4^ indicate that a child’s language development can be negatively affected by caregivers’ depression.^5,6,7,8,9^ Given that (1) women experience depressive symptoms along a wide spectrum after giving birth, (2) most mothers currently experience subclinical depressed mood in the postpartum period,^10^ and (3) subclinical levels of depressive symptoms in the early postpartum period increase the likelihood of later deterioration of mood,^11^ investigating whether subclinical levels of depressive symptoms are associated with early infant language development is important for a child’s linguistic development.

Foundations of language are already established during the first weeks after birth,^12^ making depressed maternal mood a potential risk factor associated with early language acquisition milestones. Because altered infant speech perception is associated with the risk of later language impairments,^13,14^ it is important to identify factors negatively influencing infants’ speech perception as early as possible. However, it remains unknown whether subclinical depressed mood in otherwise healthy mothers who have recently given birth is associated with early infant speech perception, and whether such an association can be detected in very early infancy.

Infant speech perception can be investigated by electrophysiological markers because electroencephalography can be performed relatively easily in young infants. The event-related potential mismatch negativity^15^ or mismatch response indicates the brain’s neural response to speech that is the response to deviant stimuli compared with the response to standard stimuli, without requiring an infant’s attention. Although mismatch negativity among adults is negative in polarity, a shift from an “immature” positive to a more adultlike mismatch response is typically observed during the first year of life.^12,16,17,18^ Of note, a delayed polarity shift of an infant’s mismatch response has been associated with the risk of developing language impairments later in life.^13,14^ Hence, the mismatch response is well suited to study trajectories of early linguistic development.

The present study investigated whether maternal mood at 2 months after birth is associated with infants’ longitudinal speech perception development. We analyzed mismatch response patterns to different acoustic speech features among infants aged 2 months and 6.5 months, to cover the age range during which a mismatch response polarity shift is expected.^12,16,17^ We hypothesized that a more depressed maternal mood during the early postpartum period is associated with a lower likelihood of developing a more mature mismatch response across infancy compared with infants of mothers with more positive mood.

## Methods

### Participants

This cohort study was conducted between January 1, 2018, and October 31, 2019. The sample, recruited from the infants database of the Max Planck Institute for Human Cognitive and Brain Sciences, Leipzig, Germany, consisted of 46 dyads of mothers and their 2-month-old infants at initial assessment. Thirty-six mother-infant dyads completed the follow-up assessment when the infants were aged 6.5 months. Infant studies of comparable sample sizes have explained mismatch response variance by factors of the learning environment, such as mismatch response variance with quantity of language input among 37 infants,^19^ mismatch response variance with amount of language exposure among 27 infants,^20^ and mismatch response variance with infant-directed speech quality among 17 infants.^21^ As we were specifically interested in maternal postpartum mood at subclinical levels, we used the German version^22^ of the Center for Epidemiologic Studies Depression Scale^23^ to screen for and exclude mothers with (past) clinical episodes of major depressive disorder. Exclusion criteria for infants were gestational age less than 37 weeks, birth weight less than 2700 g, and diagnosed hearing deficits or neurologic conditions. Parents were informed of the aim and procedure and provided written parental consent. Mothers were compensated with 7.50€ and could pick a small toy for their infants. The study followed American Psychological Association standards according to the Declaration of Helsinki^24^ and was approved by the medical faculty’s ethics committee of Leipzig University.

### Maternal Assessment

Mothers completed mood and stress questionnaires at 2 months after birth. For maternal postpartum mood, the Edinburgh Postnatal Depression Scale (EPDS)^25,26^ was used (German version^27^). Scores range from 1 to 30; higher scores indicate higher levels of depressed mood, with a cutoff of 13 points indicating a high probability of clinical depression. This cutoff ensures high specificity but lower sensitivity,^28^ as we were specifically interested in identifying mothers with subclinical levels of depressed mood. We also assessed maternal subjective perceived stress using the Perceived Stress Scale (PSS-10)^29^ (German version^30^), with higher scores indicating higher levels of perceived stress. Furthermore, mothers were screened for any psychiatric or neurologic diseases and any current medications.

### Infant Assessment

Identical protocols were used for 2-month-old and 6.5-month-old infants to assess speech perception in an electrophysiology experiment, conducted in a quiet, infant-friendly room (each appointment lasted 60 minutes). We recorded the electroencephalogram from 21 active electrodes (actiCAP system; Brain Products GmbH) at standard positions (10-20 system), referenced online to electrode Cz and a ground electrode at position Fp1. Electrooculograms were recorded from electrodes at the outer canthi of both eyes and orbital ridges of the right eye. Electrode impedances were largely less than 10 kΩ and always less than 20 kΩ. The electroencephalogram signal was amplified (BrainAmp; Brain Products GmbH), digitized online at 500 Hz, and recorded (BrainVision Recorder, version 1.21.01.02; Brain Products GmbH). During experiments, infants lay (aged 2 months) or sat (aged 6.5 months) on their parent’s lap and, if necessary, were entertained using silent toys or fed by their mothers.

For the electrophysiological assessment of speech perception, we applied a multifeature paradigm^31^ with semisynthesized syllables (eFigure 1A in the Supplement). For the standard stimulus, we used the syllable */ba/*. As deviants, we used the syllables */ga/* (consonant deviant^32,33^), */bu/* (vowel deviant^32,34^), raised the pitch by 16 Hz */ba+/* (syllable pitch [F0] deviant^35^), and lengthened the vowel by 100 ms */ba:/* (*/ba/* vs */ba:/* contrast^13^; vowel length deviant). Stimuli were recorded by a female native German speaker (16-bit sampling rate, 44.1-kHz digitization) and adjusted using Praat, version 6.0.28^36^ (a description of the stimuli is in eFigure 1A in the Supplement).

A total of 800 stimuli were presented via loudspeakers using Presentation software, version 17.2 (Neurobehavioral Systems Inc). Standard and deviant syllables were presented in alternation (eFigure 1B in the Supplement) at pseudorandom order (ie, 1 deviant type no more than twice in a row), with a total of 400 standards (probability, 50%) and 100 of each deviant syllable (probability, 12.5%). The interstimulus interval (syllable offset to onset) was 800 milliseconds (experiment length, 13 minutes).

### Electrophysiological Data Analysis

Electrophysiological data were processed using EEGLAB, version 13.5.4b^37^ and MATLAB, version R2017b (The MathWorks Inc). Offline, data were algebraically re-referenced to the mean of mastoids and bandpass filtered (windowed-sinc finite impulse response [FIR] filter, bandpass 1-30 Hz, Kaiser window, β = 7.857; filter order = 1208) and semiautomatically scanned for artifacts (criteria: abnormal values above ±100 μV, abnormal trends above a maximum slope of 100 μV/epoch and 0.5 *R*^2^ limit) for subsequent independent-component analysis. Calculated independent components^38^ were visually scanned and artifact-related components were determined, saved, and applied to continuous data bandpass filtered at 0.5 to 30 Hz (windowed-sinc FIR filter, Kaiser window, β = 7.857; filter order = 824), typically used for mismatch response analysis.^14,39^ Data epochs of 700 milliseconds postsyllable onset (prestimulus baseline, 200 milliseconds) were obtained from the corrected data and again semiautomatically scanned for artifacts. Finally, individual means for deviants (*/bu/*, */ga/*, */ba:/*, and */ba+/*) and standard (*/ba/*) were calculated, and grand means were computed.

### Statistical Analysis

Statistical analysis was performed with SPSS software, version 24 (IBM Corp) between January 1 and March 31, 2021 (initial analysis) and between July 1 and July 31, 2022 (moderation analysis). We tested for significance of the mismatch response as an indicator of infant speech perception at ages 2 and 6.5 months for all contrasts by comparing the mean event-related potential amplitude of the standard stimulus with those of the deviant stimuli at frontal (F3, Fz, and F4 electrodes),^40^ central (C3, Cz, and C4 electrodes), and posterior (P3, Pz, and P4 electrodes) regions of interest. Analyses were performed for six 100-millisecond time windows (starting 100 milliseconds after stimulus onset), while controlling for infants’ physical maturation (infant age; Table 1), which has been shown to affect infant mismatch response polarity (ie, positive vs negative mismatch response).^17^ For each age, one 3-factorial repeated-measures analysis of covariance was performed on the mean amplitude event-related potential data, with the factors condition (standard, syllable pitch, consonant, vowel, and vowel length deviant), time-window (100-200, 200-300, 300-400, 500-600, and 600-700 milliseconds), and region (frontal, central, and parietal).

**Table 1.** Overview of Maternal and Infant Characteristics

Characteristic	First assessment (n = 46)	Second assessment (n = 36)	Dropouts (n = 10)
**Maternal and family characteristics**			
Maternal age, mean (SD) [range], y	32.1 (3.8) [25-41]	32.2 (4.1) [25-41]	31.3 (2.9) [28-35]
Maternal CES-D scores, mean (SD) [range]	6.7 (5.5) [0-23]	6.2 (5.5) [0-23]	7.8 (5.9) [1-21]
Maternal EPDS scores, mean (SD) [range]	4.8 (3.6) [0-14]	4.2 (3.1) [0-11]	6.4 (4.6) [0-14]
Maternal PSS-10 scores, mean (SD) [range]	10.1 (5.8) [1-25]	9.6 (5.8) [1-23]	11.6 (5.8) [6-25]
Maternal euthyroid state while taking l-thyroxin medication, absolute No.	5	2	3
Professional qualification of mothers, No. (%)			
Without qualification	1 (2.2)	1 (2.8)	0
Completed vocational training	11 (23.9)	6 (16.7)	5 (50.0)
University student	1 (2.2)	1 (2.8)	0
University degree (or higher)	33 (71.7)	28 (77.7)	5 (50.0)
Professional qualification of fathers, No. (%)			
Completed vocational training	20 (43.5)	14 (38.9)	6 (60.0)
University student	4 (8.7)	4 (11.1)	0
University degree (or higher)	22 (47.7)	18 (50.0)	4 (40.0)
No. of older siblings, No. (%)			
None	14 (30.4)	14 (38.9)	0
1	15 (32.6)	12 (33.3)	3 (30.0)
2	4 (8.7)	3 (8.3)	1 (10.0)
No information	13 (28.3)	7 (19.4)	6 (60.0)
**Infant characteristics**			
Age, mean (SD) [range], wk	9.6 (1.2) [8-12]	28.4 (1.5) [26-32]	NA
Sex ratio, female:male	23:23	18:18	5:5
Week of pregnancy at birth, mean (SD) [range]	39.7 (1.2) [37-42]	39.8 (1.2) [37-42]	39.7 (1.2) [37-41]
Weight at birth, mean (SD) [range], g	3522.4 (375.7) [2790-4300]	3540.2 (360.9) [2820-4300]	3458.0 (439.7) [2790-4265]

Abbreviations: CES-D, Center for Epidemiologic Studies Depression Scale (critical cutoff for clinical depressive disorder, <24)^22^; EPDS, Edinburgh Postnatal Depression Scale; NA, not available; PSS-10, Perceived Stress Scale.

To investigate the association of postpartum maternal mood with longitudinal changes in infant speech perception from ages 2 to 6.5 months, moderation analyses were performed for statistically significant mismatch response effects found in the repeated-measures analyses of covariance. Within-participant factor was the mismatch response amplitude change (from ages 2 to 6.5 months) and the moderator was maternal mood (EPDS scores). Significant association of maternal mood with speech perception development from ages 2 to 6.5 months was probed by using the SPSS toolbox MEMORE^41^ and applying the Johnson-Neyman procedure^42,43^ for identifying boundaries of significance when the moderation effect of maternal mood on the change of the mismatch response amplitude from ages 2 to 6.5 months becomes significant.

Assumptions for metric statistical tests were checked; if not otherwise specified, data were normally distributed (Kolmogorov-Smirnov test). Reported *P* values are 2-sided, and effects are considered to be statistically significant at *P* < .05. *P* values were Bonferroni corrected for multiple comparisons.

### Missing Data

All infants met the criteria of at least 50% valid trials in the electrophysiological experiment. All mothers were under the clinical cutoff for a major depressive episode. From the first to second assessment, 10 infant-mother dyads withdrew.

## Results

All 46 infants (mean [SD] age, 9.6 [1.2] weeks; 23 girls and 23 boys) and 46 mothers (mean [SD] age, 32.1 [3.8]) were German monolingual, healthy, and from families with middle to high socioeconomic backgrounds; mothers did not smoke and had no history of neurotoxin use. All mothers were on maternity leave at both assessment points and reported a joint household with both parents (Table 1).

### Self-reported Maternal Mood and Perceived Stress

Table 1 provides mean (SD) EPDS scores (maternal mood) and mean (SD) PSS-10 scores (perceived maternal stress) of 46 mothers participating in the first assessment (data acquired 2 months after birth; eFigure 2 in the Supplement) as well as of 36 mothers in the final sample (data acquired 6.5 months after birth; eFigure 2 in the Supplement) who responded to our second assessment invitation. Mean (SD) EPDS scores of mothers in the final sample (4.2 [3.1]) were below the cutoff of 13.^28^ Five mothers participating in the first assessment and 2 mothers in the final sample were taking l-thyroxin and in a stable euthyroid state with this treatment. After controlling for additional factors associated with maternal mood, we did not find significant correlations between maternal EPDS scores and socioeconomic background (mother’s professional qualification [*r* = −0.19; *P* = .21] or father’s professional qualification [*r* = −0.23; *P* = .11) or number of children in the family (*r* = 0.001; *P* ≥ .99) (eTable in the Supplement). However, we found a significant correlation between maternal EPDS scores and PSS-10 scores (*r* = −0.64; *P* = .001; eTable in the Supplement). Because a mother’s perceived stress enhances clinical postpartum depression risk^44^ and is associated with 2-month-old infants’ electrophysiological patterns,^45^ we performed a moderation analysis as a control, with mismatch response amplitude change as a within-participant factor and maternal PSS-10 scores as a moderator.

### Electroencephalographic Patterns During Infant Speech Perception

**Figure 1.**
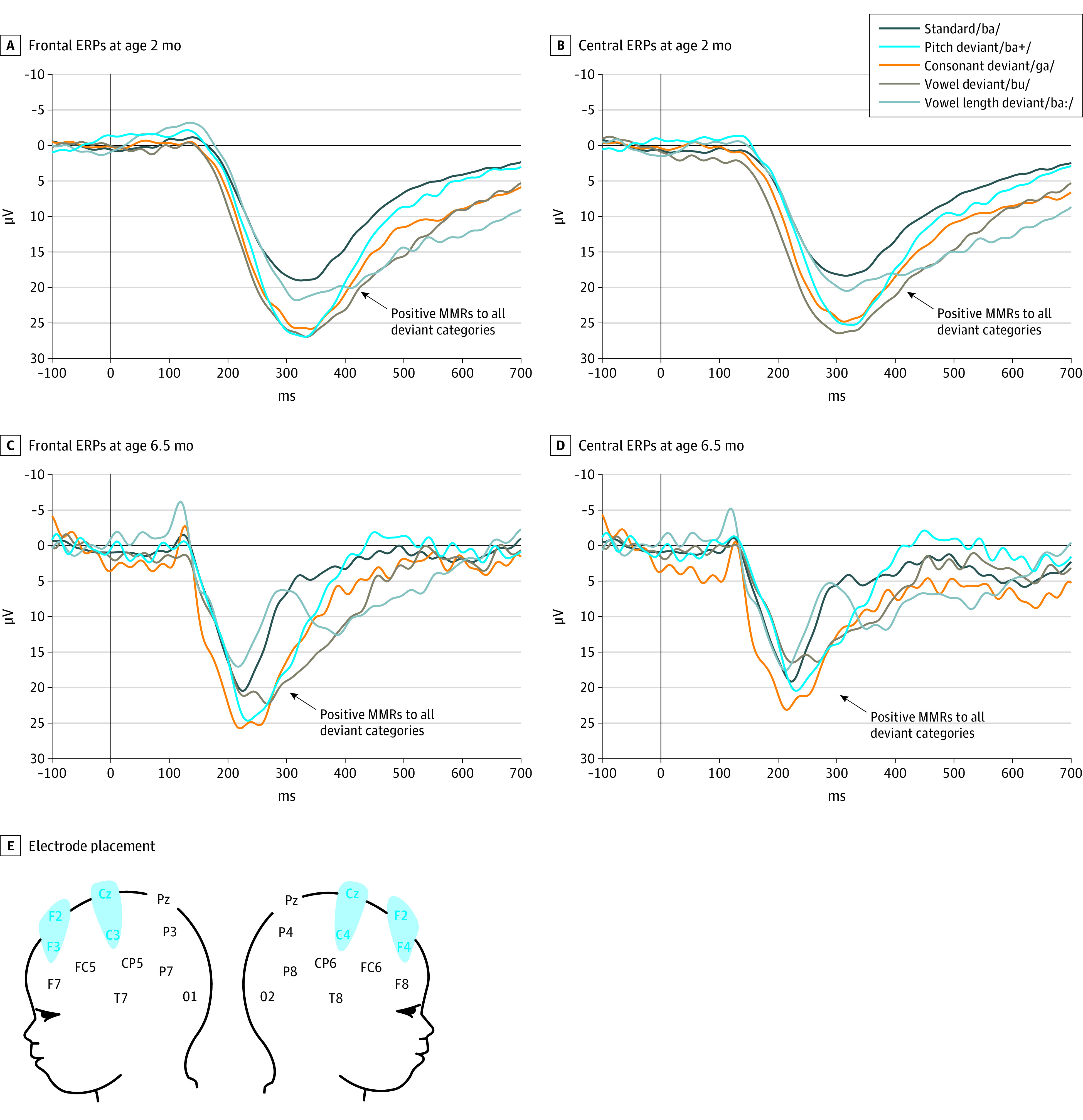
Infant Speech Perception Abilities A, Frontal event-related potentials (ERPs) to speech stimuli at age 2 months (n = 46) in response to the standard stimulus and the various deviant stimuli. B, Central ERPs to speech stimuli at age 2 months in response to the standard stimulus and the various deviant stimuli. C, Frontal ERPs to speech stimuli at age 6.5 months (n = 36) in response to the standard stimulus and the various deviant stimuli. D, Central ERPs to speech stimuli at age 6.5 months in response to the standard stimulus and the various deviant stimuli. E, Electrode placement. MMRs indicates mismatch responses.

In 2-month-old infants, a significant 3-way interaction (*R*^2^ = 0.14; 95% CI, 0.09-0.14; *P* = .003) was revealed but was not significantly associated with infant age as a control variable. Post hoc pairwise comparisons (contrasting each deviant with the standard condition at each region and time window) showed this interaction to be associated with a positive frontal mismatch response to all deviant types, as well as a positive central mismatch response to the vowel length deviant (Table 2; Figure 1A; and eFigure 3A in the Supplement).

**Table 2.** Statistical Values of the Post Hoc Pairwise Comparisons Following the Significant 3-Way Interaction Among Condition, Region, and Time Window

Deviant	Region	Time window, ms	95% CI	*P* value
**Infant aged 2 mo**				
Syllable pitch	Frontal	300-400	0.48 to 4.61	.007
Consonant	Frontal	300-400	0.20 to 4.48	.05
		400-500	0.21 to 4.49	.02
Vowel	Frontal	400-500	0.19 to 5.71	.03
Vowel length	Frontal	400-500	0.15 to 4.07	.03
		500-600	1.44 to 5.87	.001
		600-700	1.44 to 6.20	.001
	Central	500-600	1.07 to 5.69	.001
		600-700	1.23 to 6.00	.001
**Infant aged 6.5 mo**				
Syllable pitch	Frontal	200-300	1.32 to 6.46	.001
	Central	200-300	0.18 to 5.36	.03
Consonant	Frontal	200-300	1.45 to 6.42	.001
	Central	200-300	0.80 to 5.11	.002
Vowel	Frontal	200-300	2.00 to 7.39	.001
		300-400	0.76 to 6.41	.006
	Central	200-300	0.00 to 5.66	.05
Vowel length	Frontal	300-400	0.93 to 4.63	.001
		400-500	−0.06 to 4.21	.06

In 6.5-month-old infants, a significant 3-way interaction (*R*^2^ = 0.34; 95% CI, 0.29-0.35; *P* = .001) was again revealed, with no significant association of infant age with outcomes. Post hoc pairwise comparisons showed this interaction to be associated with a positive frontal mismatch response to all deviant types, as well as positive central mismatch response to the syllable pitch, consonant, and vowel deviant types (Table 2; Figure 1B; and eFigure 3B in the Supplement).

### Moderation of Maternal Mood on Infant Speech Perception Development

**Figure 2.**
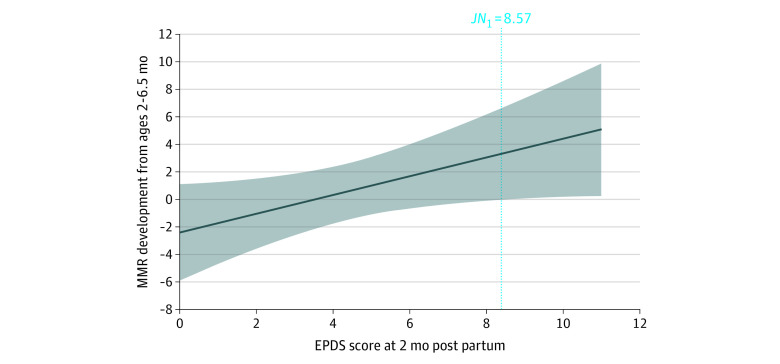
Mismatch Response (MMR) Development Between Ages 2 and 6.5 Months as a Linear Function of Maternal Mood Shown is the association of maternal mood (Edinburgh Postnatal Depression Scale [EPDS] scores) 2 months after birth with the development of infant speech perception (ie, MMR to syllable pitch changes). Higher EPDS scores are associated with less development toward a more negative MMR at age 6.5 months or even a development toward a more positive MMR amplitude. The Johnson-Neyman transition point (JN) indicates where the confidence interval around the condition effect (ie, MMR development) intersects zero on the y-axis and the association of maternal mood 2 months after birth with the development of MMR amplitude to syllable pitch changes becomes significant.

Moderation analyses of the consonant, vowel, and vowel length mismatch response did not significantly explain overall variance. We found significantly explained overall variance only for the syllable pitch mismatch response (*R^2^* = 0.12; 95% CI, 0.00-0.32; *P* = .04), with a nonsignificant intercept (coefficient, −2.41; 95% CI, −5.90 to 0.12; *P* = .08), suggesting a mismatch response change from positive to more negative amplitudes between ages 2 and 6.5 months. We also found that maternal EPDS scores were significantly associated with syllable pitch mismatch response change (coefficient, 0.68; 95% CI, 0.03-1.33; *P* = .04). Probing the moderation association of maternal mood with mismatch response development revealed that the Johnson-Neyman transition point for a significant association of maternal mood with infant speech perception change is at an EPDS score of 8.57 (Table 3; Figure 2). This finding means that mismatch response development (typically from positive to more negative values) stagnates or even becomes more positive (immature) at age 6.5 months with more depressed maternal mood and that this association becomes significant at EPDS scores of 8.57 or higher. Controlling for the association of mother’s self-perceived stress with outcomes, analysis revealed no significantly explained overall variance.

**Table 3.** Development of Infant Speech Perception (ie, Mismatch Response to Syllable Pitch Changes) at Values of the Moderator Variable Maternal Mood

Maternal mood (EPDS score)	Effect (SE) [95% CI]	*P* value
0.00	−2.41 (1.72) [−5.90 to 1.08]	.17
0.58	−2.02 (1.57) [−5.21 to 1.18]	.21
1.16	−1.62 (1.44) [−4.54 to 1.30]	.27
1.74	−1.23 (1.31) [−3.89 to 1.44]	.36
2.32	−0.83 (1.20) [−3.27 to 1.61]	.49
2.89	−0.44 (1.11) [−2.70 to 1.83]	.70
3.47	−0.04 (1.05) [−2.18 to 2.10]	.97
4.05	0.36 (1.02) [−1.71 to 2.42]	.73
4.63	0.75 (1.02) [−1.32 to 2.82]	.47
5.21	1.15 (1.05) [*−*0.99 to 3.29]	.28
5.79	1.54 (1.12) [−0.73 to 3.81]	.18
6.37	1.94 (1.20) [−0.51 to 4.39]	.12
6.95	2.33 (1.31) [−0.34 to 5.00]	.09
7.53	2.73 (1.44) [−0.20 to 6.65]	.07
8.11	3.12 (1.58) [−0.08 to 6.32]	.06
8.57	3.44 (1.69) [0.00 to 6.88]	.05
8.68	3.52 (1.72) [0.02 to 7.02]	.05
9.26	3.91 (1.87) [0.10 to 7.72]	.04
9.84	4.31 (2.03) [0.18 to 8.44]	.04
10.42	4.70 (2.20) [0.24 to 9.16]	.04
11.00	5.10 (2.36) [0.30 to 9.90]	.04

Abbreviation: EPDS, Edinburgh Postnatal Depression Scale.

## Discussion

We found that self-reported maternal mood 2 months after birth was associated with the longitudinal development of an electrophysiological indicator for infant syllable pitch perception. More depressed maternal mood was associated with a weaker maturational change of the mismatch response to the syllable pitch deviant between ages 2 and 6.5 months. This finding suggests an association between infant speech perception maturation and depressed maternal mood after birth even at a subclinical level, and a pivotal role of an early exposure to depressed mood for syllable pitch perception developmental trajectories.

Within the first year of life, the brain’s signature of processing an auditory mismatch is expected to develop from positive, immature mismatch responses to a more adultlike mismatch negativity.^12,16,17,18^ Given that this shift is regularly seen at this developmental stage, the first year of life constitutes a critical period for speech perception. This period is vulnerable to environmental influence, such as the main caretaker’s mood. Although we observed an overall maturation (less positive mismatch response) in 6.5-month-old infants compared with 2-month-old infants, infants of mothers with more depressed mood 2 months after birth showed overall more positive, less mature mismatch response polarity at both ages. This finding suggests that the main caregiver’s mood is a pace-setting factor for infant mismatch response maturation. This finding is corroborated by the observation that the developmental shift toward more negative mismatch responses at age 6.5 months when compared with age 2 months was more likely for infants of mothers with a less depressed mood. Given that infants with a prolonged period of positive “immature” mismatch responses during the first months of life are at risk of later language difficulties^12^ and immature mismatch responses to pitch variations were found among individuals experiencing language developmental difficulties,^46,47,48,49^ mothers’ more depressed postpartum mood can be considered a risk factor for infants experiencing language difficulties later in life.

Our findings extend previous reports on negative associations between maternal postpartum clinically depressed mood and children’s language development by exploring the subclinical range of maternal mood.^8,50^ They align with previous work suggesting that a reduction of infant-directed speech use among mothers with depressed mood is associated with children’s developmental trajectories.^51^ Infant-directed speech uses greater pitch variability and slower speech rate compared with adult-directed speech^52,53^ and is critical for encouraging early language perception.^54,55,56,57^ However, depressed mothers often show a reduction in vocal pitch modulation when directing speech toward their infants.^58,59,60^ Our findings lend support to the role of pitch modulations in the mother-infant relationship, even in subclinical mood deterioration: it was the response to the syllable pitch and not the other speech deviants that showed associations with infant mismatch response negativity and maternal mood. However, associations might have been missed because of the relatively small sample.

Although further studies with infant-caregiver dyads are needed to fully characterize the association of the caregiver’s mood with infant language development, the present findings suggest that the postpartum period is a critical time for infant speech perception. This finding provides the foundation for future studies testing the hypothesis that early support of mothers with postpartum depressed mood may also be beneficial for their infants’ speech perception and language development. Although pharmacologic interventions for postpartum depression have been shown to effectively encourage infant-directed speech use^59^ and to promote infant speech perception,^8^ an alternative approach of supporting women experiencing postpartum subclinical depressed mood might be the involvement of other close caregivers. As fathers also use increased pitch variability in infant-directed speech,^61^ paternal pitch variability may be one option for a low-threshold coping strategy to support infants’ language development during the postpartum period.^62^

### Limitations

This study has some limitations. The longitudinal design offers the advantage of establishing a sequence of events; however, correlation over time does not imply causation and allows only for speculation on the underpinnings of the association between postpartum maternal mood and infant syllable pitch perception. Although a reduced range in the vocal pitch of mothers with postpartum depression has been reliably shown,^58,59^ we did not test whether our main findings were associated specifically with infant-directed speech use by mothers. Future research should include assessments of mothers’ infant-directed speech characteristics to disentangle the specific aspects underlying the association between maternal postpartum mood, maternal pitch modulation, and infant mismatch response to syllable pitch deviants. Moreover, we assessed maternal mood only when infants were 2 months old. To reliably conclude that maternal mood 2 months after birth moderates less maturational change of infant speech perception from ages 2 months to 6.5 months, only a second assessment would allow for excluding that mothers continue to experience a more depressed mood, which might also be associated with infants’ speech perception at 6.5 months.

Our study offers, to our knowledge, the first evidence of an association of postpartum maternal mood at a subclinical level and infant speech perception. Future research will need to replicate these findings in a larger, more heterogeneous sample with families of broader socioeconomic backgrounds to allow for generalizability of the present results. In the present study, family-related variables such as socioeconomic background and number of siblings did not explain maternal postpartum mood. However, even in our limited sample with mostly middle to high socioeconomic status, the observed variance in mood showed an association with infant speech perception.

## Conclusions

The results of this cohort study suggest an association of postpartum depressed maternal mood at subclinical levels with less maturational change of speech perception among young infants between ages 2 and 6.5 months. Our observation that maternal mood at 2 months post partum was associated with infant speech perception longitudinally emphasizes the importance of considering the role of postpartum maternal mood in shaping infants’ brain responses to speech stimuli. This finding advocates for systematic early screenings for mothers’ postpartum mood and for future studies exploring whether early support for caregivers experiencing depressed mood can have a positive association with infant language development.

## Notes

Supplement. **eFigure 1.** Description of Experimental Speech Stimuli **eFigure 2.** Description of Experimental Protocol **eTable.** Correlation of Maternal Mood (EPDS-Scores) With Mother’s and Father’s Professional Qualifications, Number of Children in the Family and Mother’s Perceived Stress **eFigure 3.** Topographic Maps of the Mismatch Response
