# Supplementary material for: Association of Postpartum Maternal Mood With Infant Speech Perception at 2 and 6.5 Months of Age
Source: JAMA Netw Open. 2022 Sep 21;5(9):e2232672. doi: 10.1001/jamanetworkopen.2022.32672 (PMC9494190; doi:10.1001/jamanetworkopen.2022.32672)
Supplement: Supplement. — eFigure 1. Description of Experimental Speech Stimuli eFigure 2. Description of Experimental Protocol eTable. Correlation of Maternal Mood (EPDS-Scores) With Mother’s and Father’s Professional Qualifications, Number of Children in the Family and Mother’s Perceived Stress eFigure 3. Topographic Maps of the Mismatch Response [file jamanetwopen-e2232672-s001.pdf]

Supplementary Online Content

Schaadt G, Zsido RG, Villringer A, Obrig H, Männel C, Sacher J. Association of postpartum maternal mood with infant speech perception at 2 and 6.5 months of age. *JAMA Netw Open.* 2022;5(9):e2232672. doi:10.1001/jamanetworkopen.2022.32672

eFigure 1. Description of Experimental Speech Stimuli

eFigure 2. Description of Experimental Protocol

eTable. Correlation of Maternal Mood (EPDS-Scores) With Mother's and Father's Professional Qualifications, Number of Children in the Family and Mother's Perceived Stress

eFigure 3. Topographic Maps of the Mismatch Response

This supplementary material has been provided by the authors to give readers additional information about their work.

© 2022 Schaadt G et al. *JAMA Network Open.*

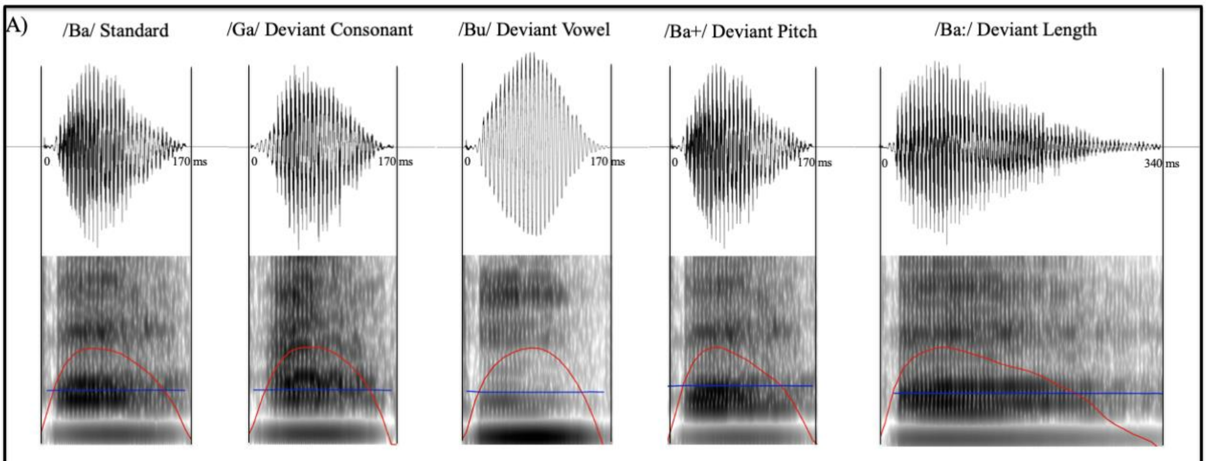

Information on acoustic parameters, as well as other stimulus characteristics of the standard syllable and deviant syllables

Acoustic parameters	Standard /ba/	Deviant /ga/	Deviant /bu/	Deviant /ba:/	Deviant /ba+/
F0	198.00	198.00	198.00	198.00	214.00
1st Formant (Hz)	1145.53	918.24	350.33	1100.41	1145.53
2nd Formant (Hz)	1497.10	1814.13	1264.55	1499.30	1497.10
3rd Formant (Hz)	3032.04	3039.53	3030.41	3036.44	3032.04
4th Formant (HZ)	4217.78	4215.47	4188.21	4212.64	4217.78
Length in ms*	170	170	170	270	170
Mean Intensity#	70	70	70	70	70

* Additionally, a silent period of 50 ms before and after each syllable was added, # All stimuli were set to the same intensity and normalized to a mean intensity db Sound Pressure Level

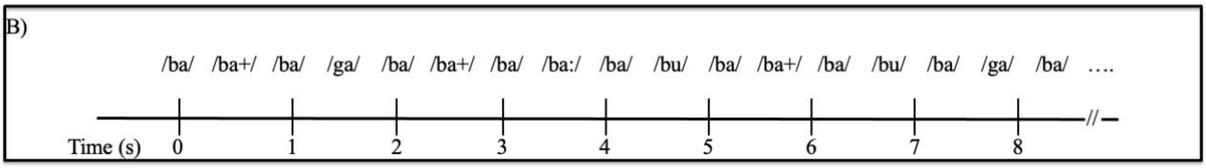

eFigure 1. Description of Experimental Speech Stimuli. **(A)** Acoustic parameters. Illustrated are the acoustic parameters of the standard syllable /ba/ and the four different deviant stimuli: consonant change /ga/, vowel change /bu/, syllable-pitch change /ba+/, and vowel length change /ba:/. In the spectrograms, the red line illustrates the intensity contour, and the blue line illustrates the pitch contour. The table gives information on the *mean* of the fundamental frequency (pitch), the higher order Formants (first to fourth), length and intensity. **(B)** Experimental structure of the multi-feature paradigm. The standard stimulus and the different deviant stimuli are alternatingly presented.

© 2022 Schaadt G et al. *JAMA Network Open.*

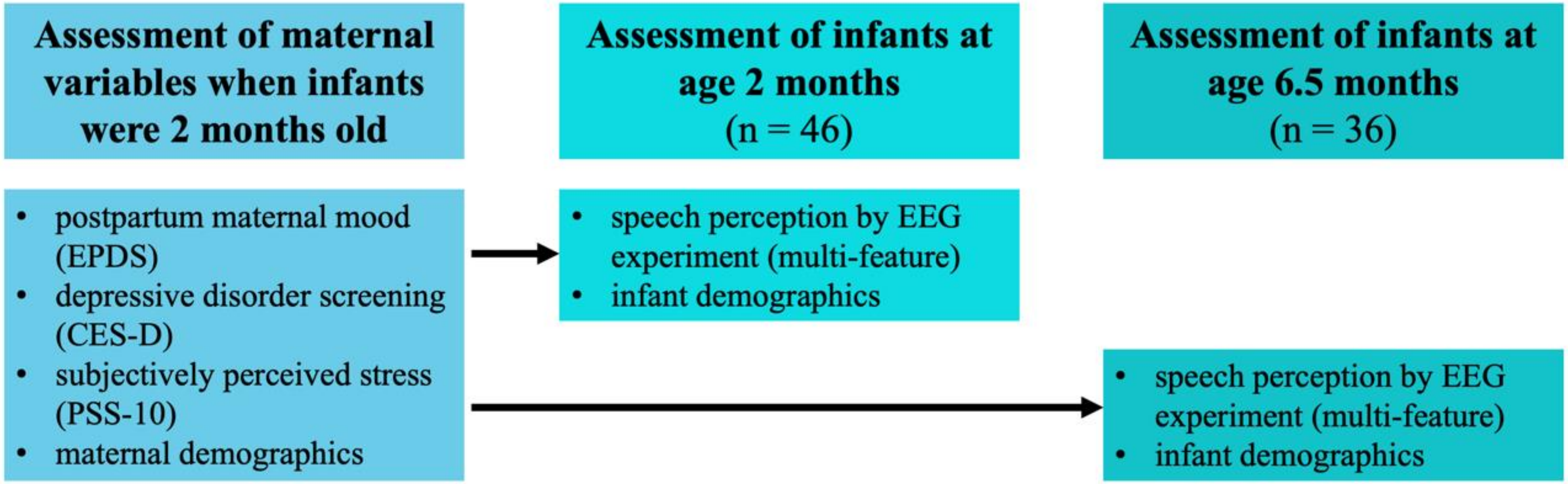

eFigure 2. Description of Experimental Protocol. Illustrated are the assessments of maternal and infant variables. Maternal variables were assessed when infants were 2-months old (maternal mood: EPDS, CES-D, PSS-1; age). The associations between maternal mood and infant speech perception at age 2-months and 6.5-months were analyzed. For the first infant assessment at age 2-months (n=46), infant speech perception and infant demographics (age, sex, birth weight, week of pregnancy at birth) were assessed. For the second assessment (n=36) at age 6.5-months, only infant speech perception and infant age were assessed (with 10 dropouts). EEG = Electroencephalography; EPDS = Edinburgh Postnatal Depression Scale; CES-D = Centre for Epidemiologic Studies Depression Scale; PSS-10 = Perceived Stress Scale.

© 2022 Schaadt G et al. *JAMA Network Open.*

eTable. Correlation of Maternal Mood (EPDS-Scores) With Mother's and Father's Professional Qualifications, Number of Children in the Family and Mother's Perceived Stress

	Mother's professional qualification	Father's professional qualification	Number of children in the family	PSS-10
EPDS	*r*= –0.19; *P*=.21	*r*= –0.23; *P*=.11	*r*=0.001; *P*=.99	*r*= 0.64; *P*=.001
Note. EPDS = Edinburgh Postnatal Depression Scale; PSS-10 = Perceived Stress Scale.				

© 2022 Schaadt G et al. *JAMA Network Open.*

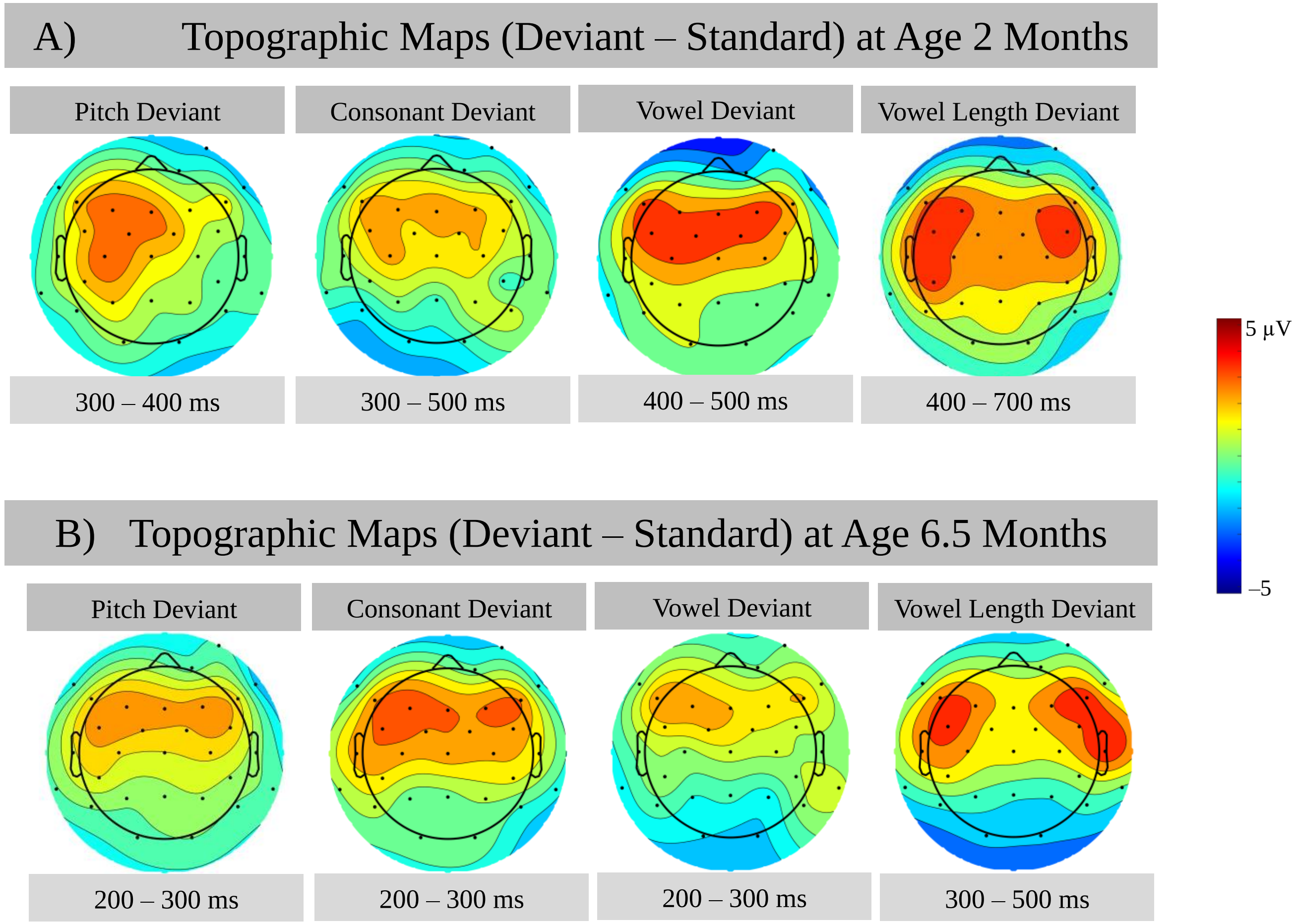

eFigure 3. Topographic Maps of the Mismatch Response (MMR). **(A)** Illustrated is the MMR (deviant – standard) for all deviant categories in those time windows, where significant MMRs were elicited (note that time windows with significant effects differ depending on the deviant) at age 2-months. **(B)** Illustrated is the MMR (deviant – standard) for all deviant categories in those time windows, where significant MMRs were elicited (note that time windows with significant effects differ depending on the deviant) at age 6.5-months.

© 2022 Schaadt G et al. *JAMA Network Open.*
